# Replications, Comparisons, Sampling and the Problem of Representativeness in Animal Cognition Research

**DOI:** 10.26451/abc.08.02.14.2021

**Published:** 2021-05

**Authors:** Benjamin G. Farrar, Konstantinos Voudouris, Nicola S. Clayton

**Affiliations:** Department of Psychology, University of Cambridge

**Keywords:** Animal cognition, Comparison, Experimental design, Generalizability, Replication, Representativeness, Sampling

## Abstract

Animal cognition research often involves small and idiosyncratic samples. This can constrain the generalizability and replicability of a study’s results and prevent meaningful comparisons between samples. However, there is little consensus about what makes a strong replication or comparison in animal research. We apply a resampling definition of replication to answer these questions in Part 1 of this article, and, in Part 2, we focus on the problem of representativeness in animal research. Through a case study and a simulation study, we highlight how and when representativeness may be an issue in animal behavior and cognition research and show how the representativeness problems can be viewed through the lenses of, i) replicability, ii) generalizability and external validity, iii) pseudoreplication and, iv) theory testing. Next, we discuss when and how researchers can improve their ability to learn from small sample research through, i) increasing heterogeneity in experimental design, ii) increasing homogeneity in experimental design, and, iii) statistically modeling variation. Finally, we describe how the strongest solutions will vary depending on the goals and resources of individual research programs and discuss some barriers towards implementing them.

Animal cognition research often involves small samples. In order to make general claims about a group or species’ behavior, researchers assume that their samples are representative enough of the group or species of interest. However, this assumption is rarely tested, and the literature is populated by claims that are produced by single laboratories, testing the same animals, at single time points and in closely related experimental designs. This could lead to overgeneralized findings that are difficult to replicate (Baker, 2016; Henrich et al., 2010; Würbel, 2000; Yarkoni, 2019), but equally, it could be an effective strategy to maximize scientific progress in resource-limited fields (Craig & Abramson, 2018; Davies & Gray, 2015; Mook, 1983; Schank & Koehnle, 2009; Smith & Little, 2018). To explore this issue, this article shows how concerns about replicability, representativeness, comparison and theory testing, and pseudoreplication are all related through the lens of sampling. To design the best experiments, researchers should consider all five in relation to their sampling plans. Part 1 of this article focuses on sampling and replication, and answers the following questions:

- What is a replication in animal behavior and cognition research?
- What is the relationship between replication and theory testing?
- What makes a species-fair comparison?

Part 2 of the article then focuses on representativeness and asks how concerned researchers should be with the problem of non-representative sampling in animal research. We explore this issue through a re‐analysis of existing data on animal ‘self-control’ and a simulation study. The simulation study shows that, for some between-group or between-species comparisons, poorly representative samples could lead to false positive rates closer to 50% than 5%, the rate conventionally cited when authors use *p* < .05 to define statistical significance. Finally, we end the article with a discussion of how researchers might assess, mitigate, and account for the problem of representativeness in comparative cognition.

## Part 1 – Claims, Samples and Replications

### What Are Replications in Animal Research?

A study is labeled a replication because it is the same in some regards to a previous experiment. For example, a replication study may repeat the same experimental protocol as a previous study, except use a new sample of animals. However, it is not possible to perform *exactly* the same study twice, and because of this, any replication study can also be reframed in terms of a test of generalization. Even if the same experimenters perform the same experiment on the same group of animals, the replication experiment is still a test of generalization across time.

However, while truly identical replications are impossible, this does not mean the concept of replication is obsolete, or redundant with generalizability. When performing replications, scientists are not usually interested in what philosophers call absolute identity, but in what they call relative identity (Geach, 1973; Lewis, 1993; Noonan & Curtis, 2004; Quine, 1950). They are not interested in whether a feature of a replication is exactly the same as an original study, rather, they are interested in whether that feature *can be considered* the same, or as coming from the same population, relative to a given theory. Idealistically, a theory or claim would specify what can and cannot be considered as coming from the same population, i.e., identifying its boundary conditions (e.g., Simons et al., 2017), and thus what a valid test of it would sample from. For example, consider the Rescorla-Wagner model, which specifies that gains in associative strength are proportional to the prediction error (Rescorla & Wagner, 1972). From the perspective of the Rescorla-Wagner model, it does not matter whether the hypothesis is tested with a sample of rats or a sample of mice, or pigeons, or monkeys, etc. Providing a valid conditioning procedure is followed, all of these species are within the boundary conditions of the Rescorla-Wagner model, and an original study making a general claim about the Rescorla-Wagner model by testing rats could therefore be replicated in pigeons or in monkeys – the Rescorla-Wagner model makes no distinction. On the contrary, the most robust tests of the Rescorla-Wagner model would sample from across all of species that the model applies to, rather than just a single species.

Recently, resampling definitions of replication have been developed (Asendorpf et al., 2013; Machery, 2020). These may be the most effective definition of replication in animal cognition research. When researchers test a claim, they sample from populations of experimental units (most often animals), settings, treatments, and measurements (Gómez et al., 2010). For example, when testing the claim that chimpanzees will explore a mark on their forehead when exposed to a mirror, researchers sample from the population of chimpanzees available for research, from various settings (laboratories, zoos, wild), with a variety of possible treatments (different size mirrors, different types of marks, etc.), and many different possible measurements (e.g., an ethogram of self-directed actions). The resampling definition of replication states that a replication study is a study that resamples from the same populations of experimental units, treatments, measurements, and settings that an original study could have sampled from, relative to the claim being tested (Machery, 2020; Nosek & Errington, 2020). This is outlined in Table 1, which is adapted from Machery (2020).

According to the resampling approach, a complete replication resamples from the same populations of experimental units, treatments, measurements, and settings as an original study, relative to the theory or claim in question. However, an experiment could also replicate some features of an original study but not others (Machery, 2020). This would create an explicit test of generalizability; probing whether the claim or theory can be applied successfully outside of some of its pre-specified boundary conditions. For example, a researcher would be able to test whether theories built on work with captive monkeys generalize to their wild counterparts by resampling from the *same* population of, treatments and measurements, but sampling from a *different* population of settings (captive versus wild).

To see how the resampling definition can be applied in animal cognition research, we now discuss a partial or “conceptual” replication of a study investigating aging in monkeys (Almeling et al., 2016; Bliss-Moreau & Baxter, 2019). This is a useful example as, like most experiments in animal cognition, Bliss-Moreau and Baxter’s study is not a close replication of the previous study; it was neither conducted in identical laboratory settings nor even in the same model species.

### Case Study: Do Nonhuman Primates Lose Interest in the Non-Social World with Age?

In 2016, Almeling and colleagues examined the relationship between the age of monkeys and their interest in the social and non-social environment. They tested 116 Barbary macaques housed in a large (20 ha) outdoor park in France. Across three non-social novel object interest tasks, Almeling et al. reported that older Barbary macaques interacted less with objects compared to younger Barbary macaques (*N* = 88 in these tasks). From this, they made the general claim that nonhuman primates lose interest in the non-social world with age. Bliss-Moreau and Baxter (2019) replicated one of the object conditions of Almeling et al. in a larger sample of 243 rhesus macaques. However, these rhesus macaques were housed in indoor cages either alone or with a social pair mate, in contrast to the free‐roaming Barbary macaques. Bliss-Moreau and Baxter labeled their study as a “conceptual” replication because they tested a different species in a markedly different setting and used a different, albeit conceptually similar, food-baited apparatus. However, relative to the claim that monkeys, in general, display a loss of interest to non-social stimuli with age, the populations sampled by Bliss-Moreau and Baxter do seem to come from the same overall populations that Almeling et al.’s claim specifies, i.e., both are tests of the claim that interest in the non-social environment declines during aging in monkeys.

Bliss-Moreau and Baxter reported no statistically significant effect of age on exploration across the first two minutes, which they interpreted as contrary to the results of Almeling et al. (2016) and challenging “the notion that interest in the ‘non-social world’ declines with age in macaque monkeys, generally” (Bliss-Moreau & Baxter, 2019, p. 6). This claim seems reasonable: both Almeling et al. and Bliss-Moreau and Baxter sampled from within the experimental units, setting, treatment, and measurement populations implicitly specified by the claim that interest in the non-social world declines with age in macaque monkeys, and so our confidence in the claim overall should decrease, following the negative replication results. But can we really say that Bliss-Moreau and Baxter’s experiment *replicated* Almeling et al.’s? This question is difficult, because replications exist on many levels (across experimental units, settings, treatments, and measurements) and are theory or claim dependent. Moreover, most experiments in animal behaviour and cognition do not make a single isolated claim. For example, the following theoretical claims could reasonably be inferred from the Almeling et al. paper:

1) Socially living Barbary macaques lose interest in the non-social environment with age
2) Barbary macaques lose interest in the non-social environment with age
3) Socially living monkeys lose interest in the non-social environment with age
4) Monkeys lose interest in the non-social environment with age

When asking how Bliss-Moreau and Baxter’s (2019) study is a replication of Almeling et al.’s, (2016) we should consider not just how the studies relate to each other, but how they relate to each claim we are assessing. Ultimately, the goal of a replication study is usually to test a scientific claim, rather than just to match a previous study’s methods (Nosek & Errington, 2020). Therefore, when interpreting the results of replication studies, researchers should focus on how relevant and diagnostic the data from each study are to the claim(s) in question, rather than just how similar they are. The main strength of the resampling definition of replication — that a replication study resamples from the same populations that an original study could have sampled from, relative to the claim being tested — is that it forces researchers analyzing replication studies to consider exactly what is being tested and how effective the test is, rather than focusing unnecessarily on absolute similarity.

One barrier to identifying and testing claims is that many theories and claims in animal cognition are verbal and vague. This makes it difficult to derive risky predictions of the theories, because their vagueness affords them the flexibility to accommodate nearly any result (Roberts & Pashler, 2000). This could be remedied by formally modelling theories and hypotheses (Farrell & Lewandowsky, 2010; Guest & Martin, 2020), and some suggest these models are key to making progress in understanding animal minds (Allen, 2014), or in understanding what comparative cognition can achieve as a science (Farrar & Ostojić, 2019). These models can be informed by known mechanisms driving animal behavior, such as associative learning (Heyes & Dickinson, 1990; Lind, 2018; Lind et al., 2019), but these need not be preferred to, or even contradict, non-associative models (Bausman & Halina, 2018; Mercado, 2016; Smith et al., 2016). Just like any other scientific tool, formal models need critique from a variety of perspectives; but the benefit of these models is that they facilitate such critique, in comparison with verbal theories that can avoid it.

### Species-Fair Comparisons

The resampling account not only offers a theoretical framework for replications, generalizations, and theory testing in animal cognition research, but it also offers a framework for analyzing between-species comparisons. Between-species comparisons are just tests of the generalizability of an effect across species, and like any other test of generalization, they can be reframed in terms of replication, too. Comparing an effect between a group of chimpanzees and a group of bonobos is the same as testing if the effect generalizes from chimpanzees to bonobos, or replicating a study from chimpanzees in bonobos, and both of these are entailed by a coarser study of whether great apes (chimpanzees, bonobos and orangutans) show the effect in question. Whether the study in question is best described as a comparison, replication or a test of a claim is somewhat moot — it is all three at the same time, relative to claims of different coarseness.

However, there are clearly times when researchers may wish to focus on comparative claims, and this requires sampling from *different* population of experimental units, e.g., different breeds, groups, or species of animals (with the caveat that these could be seen as coming from the same population relative to broader claims). For an ideal comparison between two groups of animals, researchers would sample from different populations of experimental units, and the same populations of treatments, measurements, and settings. Again, “same” here does not mean identical, but the same relative to the claim and experimental unit at hand. For example, consider a researcher who wants to compare the relative response of dolphins (e.g., Hill et al., 2016) to familiar and unfamiliar humans with that of elephants (e.g., Polla et al., 2018). Clearly, the researcher must sample from different populations of experimental units [dolphins, elephants], and a different population of settings [aquatic, non-aquatic]. However, even though the settings are different in absolute terms, they are the same relative to the experimental unit; the dolphins are tested in water, the elephants on land, and this makes the comparison more valid (Clark et al., 2019; Leavens et al., 2019; Tomasello & Call, 2008), or a ‘species-fair’ comparison (Boesch, 2007; Brosnan et al., 2013; Eaton et al., 2018; Tomasello & Call, 2008).

## Part 2 – The Problem of Representativeness in Animal Research

A sampling perspective shines light on why many results in animal research may struggle to replicate. Animal experiments often sample a small number of animals at a single site, using a single apparatus and measurement technique. However, from these small samples come general claims about animal behavior, creating a mismatch between the statistical model and the theoretical claim (Yarkoni, 2019). The statistical model will usually allow generalization to the population that the experimental units were randomly sampled from, for example the population of animals at a given site, (although even then they may not be randomly sampled, see Schubiger et al., 2019), but any inferences to the wider population of interest will be overconfident, unless the population of interest can be justified as the individual animal (Smith & Little, 2018). This is an unavoidable consequence of working with difficult to reach populations (Lange, 2019), but it should be accounted for when building theories. This is important as many aspects of animal behavior vary across samples; for example, due to experimenter effects (Beran, 2012; Bohlen et al., 2014; Cibulski et al., 2014; Pfungst, 2018; Sorge et al., 2014), genetic variation (Fawcett et al., 2014; Johnson et al., 2015; MacLean et al., 2019), housing conditions (Farmer et al., 2019; Hemmer et al., 2019; Würbel, 2001), diets (Davidson et al., 2018; Höttges et al., 2019), and learning/developmental histories (Skinner, 1976).

### Situating the Problem of Representativeness

The problem of representativeness has been discussed from several different angles across scientific literatures, unfortunately poorly connected and with different terminologies. However, they share the similar underlying concern that researchers’ claims are poorly matched by their sampling strategies and statistical models.

#### Replicability

First, a lack of representative sampling causes low replicability or results reproducibility (not to be confused with computational reproducibility, e.g., see Culina, van den Berg et al., 2020; Minocher et al., 2020): because of small and non‐representative samples of experimental units, settings, treatments, and measurements, sampling variation will mean that laboratories will struggle to replicate or reproduce the results of previous experiments. This argument has featured heavily in rodent phenotyping studies (Crabbe et al., 1999; Kafkafi et al., 2017, 2018; Lewejohann et al., 2006; Richter et al., 2009, 2010, 2011; Wahlsten et al., 2003; Würbel, 2000, 2002).

#### Generalizability and External Validity

Second, a lack of representative sampling causes problems of generalizability or external validity: researchers’ claims will not often generalize to novel but related settings (Yarkoni, 2019).

#### Pseudoreplication

Third, the lack of representative sampling in animal research is usually due to non-random sampling from the population of interest. This leads to pseudoreplication (Hurlbert, 1984; Lazic, 2010) if this non‐random sampling is not accounted for in the statistical models, and the consequence is that uncertainty intervals will be overly narrow, and the results will struggle to replicate in new samples – or generalize to them.

#### Theory Testing

Fourth, the lack of representative sampling produces weak tests of a theory or claim (Baribault et al., 2018): a test that probes only a small sample space of a theory’s predictions provides less opportunity for weaknesses in the theory or claim to be found, compared to a test that covers most of the relevant sample space.

### The Difficulty of Identifying Differences Between Groups and Species

That animal behavior differs across space and time makes it difficult to understand whether species or group differences in behavior are really a consequence of real species differences, or whether they are due to the host of other factors that vary between sites. In reality, the observed differences between two groups will be the sum of the real group differences in behavior that are of interest and all other factors that influence animal behavior and vary between sites. When making quantitative between-species and between-group comparisons, they are nearly always confounded by site-specific differences in factors that are not the focus of interest. Lazic (2016) commented on such a scenario in an introductory textbook for laboratory biology: “To make valid inferences, one would need to assume that the effects of [site] are zero. Moreover, as this assumption cannot be checked, the researcher can only hope that [site] effects are absent. Such a design should be avoided” (Lazic, 2016, p. 68).

One may object to this and acknowledge that, while there are many variables that differ between sites but go unmeasured, the net sum of these effects should be close to zero across sites, i.e., they will cancel each other out. However, this would only be the case if there were many variables with small effect that were randomly assigned to each site, and this is not what happens. On the contrary, laboratories or sites differ markedly from each other on a range of variables with large effects (e.g., housing conditions, learning experiences). It is often recognized that animal laboratories are poorly positioned to generate representative data of the species in the wild (Boesch, 2020; Calisi & Bentley, 2009), but what if they are also poorly positioned to generate representative data of the species in laboratories? Taken to the extreme, there may be a laboratory that is testing a sample that is more representative of a species other than its own; for example, a sample of lemurs that have parrot-like self-control, or a sample of handreared wolves that behave more like dolphins when presented with a novel object. To highlight the difficulties of making between group or between species inferences across sites, we now consider a case study of between species comparisons made using the cylinder task, and then present a simulation study of how sampling affects comparisons in animal research.

### Case Study: Between Species Comparisons and the Cylinder Task

**Figure 1:**
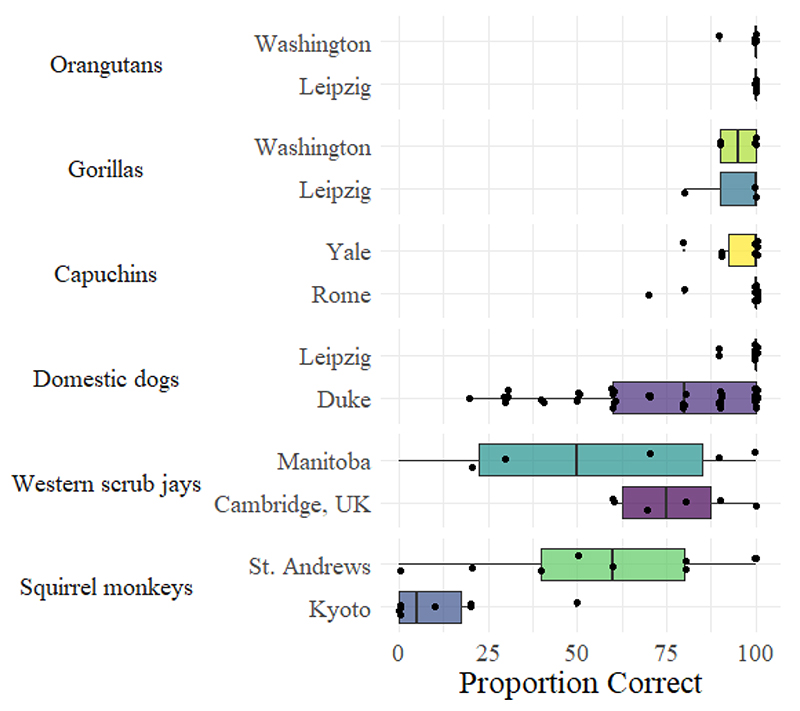
Species Differences Between Sites in the Cylinder Task *Note.* All data from MacLean et al. (2014), except the Manitoba scrub-jay data, which are from Stow et al. (2018).

For this case study, we used data from MacLean et al. (2014) to probe the stability of a measurement of behavioral inhibition when taking new samples of experimental units at new sites. MacLean et al.’s (2014) large-scale study tested the performance of 36 species across 43 sites on two tasks aimed at measuring self-control (but rather measured one form of behavioral inhibition: Beran, 2015); the A not B task and the cylinder task. The cylinder task was given to 32 species across 38 sites. In this task, animals are familiarized with retrieving a piece of food from the center of an opaque cylinder. After retrieving the food from the opaque cylinder in 4 out of 5 consecutive training trials, the animals proceed to testing. In testing, the animal is presented with a transparent cylinder with food in the center. In order to successfully retrieve the food, the animal needs to inhibit an initial drive to directly reach for the food which would cause them to subsequently collide with the transparent cylinder, and instead detour to the cylinder ends to access the food. Each animal was given 10 trials, and an overall score between 0% (no animals succeeded on any trial) and 100% (all animals succeeded on every trial) was computed for each species. Five species (orangutans, gorillas, capuchin monkeys, squirrel monkeys and domestic dogs) were tested across two sites. Figure 1 displays the between-site variation for these species, and also includes data from an additional species, the Western scrub-jay, that was tested both in the original experiment and a couple of years later at a new site (Stow et al., 2018).

For the species not performing near ceiling (scrub-jays, squirrel monkeys and domestic dogs), the variability is striking. For squirrel monkeys, the median score in Kyoto was 5%, compared with 60% in St Andrews. No individual in Kyoto performed above the median in St Andrews, and this demonstrates how some between-site differences that cannot be attributed to species identity can have large influences on behavior. To highlight the issues this can pose for inference, consider what would happen if the animals from Kyoto were not squirrel monkeys, but Tonkean macaques. Then, it is likely that the difference in performance compared to the St Andrews’ squirrel monkeys would likely be interpreted as a species difference – “Tonkean macaques are worse at behavioral inhibition than squirrel monkeys,” could be the title of a paper reporting these results. In fact, the substantial difference in behavior between species tested at different sites need not imply meaningful species differences at all. If we took new samples for all species that MacLean et al. tested, it is possible a completely different ranking of animals would be produced. MacLean et al.’s (2014) overall model gains credibility, however, because of the use of phylogenetic models (and also including data from the A-not-B task, another test of behavioral inhibition). Incorporating phylogeny and estimating phylogenetic signal when making comparisons, providing there is enough data, little bias, and sufficient model checks, can lead to large increases in statistical power (Freckleton, 2009; see MacLean et al., 2012 also for an overview of other benefits of comparative phylogenetic models). However, any individual site comparison of non-ceiling cylinder task performance between species, either within the MacLean et al. study, or from other published research, is likely too uncertain to produce meaningful estimates at the species level, and this can lead researchers astray when making inferences from individual results. Table 2 presents some statements from studies that followed MacLean et al.’s procedures using a single species at a single site, along with the species’ cylinder task “score.”

This set of numerical comparisons are factually correct, but what do they mean? The worst performing chickens actually scored higher than the Kyoto squirrel monkeys, and if we sampled another population of great tits it is possible that their performance would regress close to the mean value of all species. Ordaining a species with a single score following a single test on a small sample of animals from a single site with a single apparatus, and then comparing this number between species has no means of error control and hides the uncertainty in their estimates. Several of the inferences are reasonable; for example, we may genuinely believe that chickens will perform poorly on behavioral inhibition tasks, but this is primarily constrained by our (arbitrary) prior beliefs. For potentially more surprising results, such as the high score of great tits, our beliefs are not so constraining, yet neither are the data.

Moreover, and counter-intuitively, the best estimate of great tit performance on the cylinder task is not the 80% reported by Isaksson et al. (2018), even though this is the only known data collected with great tits on this task. Rather, the best estimate would utilize the information we have about similar animals (other birds of a similar size/socio-ecology/phylogeny), that would shrink our estimate of great tit performance closer to the mean value for, as an example, all Passeriformes tested to date. Interestingly, during the revision process of this article, two further datasets of great tit performance on the cylinder task became available. In contrast to the 80% reported by Isaksson, and in line with our prediction of regression to the mean, Troisi et al. (2020) recorded a score of 38%, and a sample of 35 tested by Coomes et al. (2020) scored 41%. Moreover, in a pilot to one of these studies using a larger tube, a sample of great tits scored 0%, suggesting that the size of the tube can heavily modulate individual’s performance (G. L. Davidson, personal communication, Jan., 2021).

How, then, can we make better inferences from single site samples of data? We could attempt to get a better estimate at this single site; for example, by testing great tits on a wide range of tube apparatuses. Alternatively, we can also use the data from other species to inform our great tit estimate. Because the behaviour of different animals will often be correlated, for example as a function of phylogenetic distance, we should allow data from similar species to guide each other’s estimates. Ideally, a phylogenetic model would be constructed that incorporates information on the phylogenetic distance between species and a model of the trait’s evolution (McElreath, 2016). Other relevant predictor variables, such as body size, tube size, or body size/tube size ratio, could be added into these models, also, or they could be investigated in separate meta-regression models. However, for many animal cognition questions, such models will be difficult to generate, but the general principle holds: when a surprisingly high or surprisingly low estimate of a species behavior is produced, and most data from similar species are less extreme, it is likely that the new estimate is over- or underestimated. Returning to the cylinder task, it is clear that non-ceiling results are not very informative about animal cognition if we do not know whether the results from any given sample are stable across space or time — before considering issues of construct validity (Beran, 2015; Kabadayi et al., 2017, 2018).

### Simulation Study

To illustrate how between-site variation (a proxy for the sum of setting, treatment and measurement variation) can lead to elevated false positive rates and results that struggle to replicate, we now present a short simulation study of a replication and a comparison in comparative cognition. The simulation and visualizations were performed in R 4.0.2 (R Core Team, 2020), using the packages *tidyverse* (Wickham et al., 2019), *extrafont* 0.17 (Chang, 2014) and *scales* 1.1.1 (Wickham & Seidel, 2020). The code is available at: https://github.com/BGFarrar/Replications-Comparisons-and-Sampling. This section can be skipped if the reader is already comfortable with the topic. We simulated a hypothetical within-species replication between two groups of chimpanzees, and a hypothetical between-species replication/comparisons between a group of chimpanzees and a group of bonobos. We simulated 100 hypothetical sites of chimpanzees, and 100 hypothetical sites of bonobos, with 100 animals at each site. The behavior of animals within a site was correlated, such that animals sampled from the same sites, on average, had more similar behaviors than animals sampled from different sites. At each site, we ‘measured’ each animal’s behavior to produce a neophobia and self-control score for each. For both the replication simulation and the comparison simulation, four parameters were used to simulate each animal’s behavior: a population grand mean, *β_0_*, a by-location random intercept *L_0_* a by-subject random intercept *S_0_*, and a by-individual residual error term *e_ls_*. Subject was nested within location, such that all subjects at the same location had the same location effect. Data were simulated using the following formula:

$${\text{Score}}_{ls}=\beta_{0}+L_{0l}+S_{0s}+e_{ls}$$

For the replication simulation, 10,000 chimpanzees were simulated with the following settings:

$$\begin{array}{l} \text{Neophobia}\\ \beta_{0}=800\\ L_{0l}∼N(0,100)\\ S_{0s}∼N(0,100)\\ e_{ls}∼N(0,50) \end{array}$$

$$\begin{array}{c} \text{Self-control}\\ \beta_{0}=80\\ L_{0l}∼N(0,10)\\ S_{0s}∼N(0,10)\\ e_{ls}∼N(0,5) \end{array}$$

**Figure 2:**
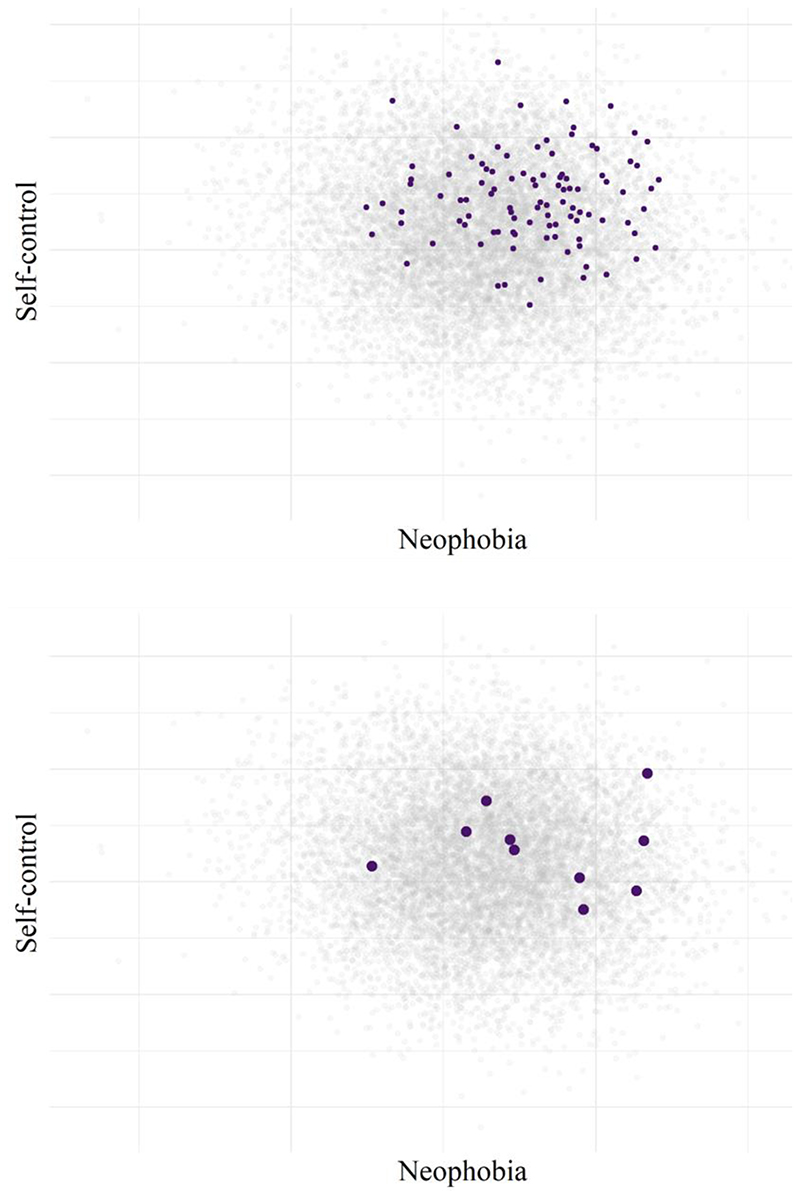
The Behavior-Space of a Simulated Population of 10,000 Chimpanzees (grey dots in both panels). *Note.* In purple, the Upper Panel shows 100 hypothetical chimpanzees sampled from a single site, and the Lower Panel shows just 10 of these chimpanzees.

The panels of Figure 2 display the behavior of all 10,000 chimpanzees (100 animals x 100 sites) in grey. Next, we randomly selected one site to be our first sample. The upper panel of Figure 2 highlights all 100 chimpanzees from this site. However, in reality we would not usually have access to or test 100 animals at a site; instead, a primate cognition sample size is usually around 7 (Many Primates, Altschul, Beran, Bohn, Caspar et al., 2019). Therefore, we randomly selected 10 animals, which are highlighted in the lower panel of Figure 2.

**Figure 3:**
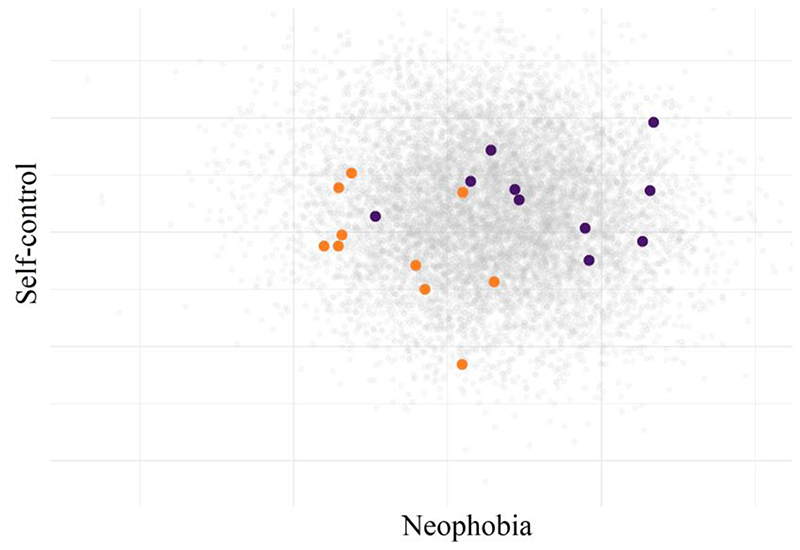
A Hypothetical Within-Species Replication, or Between-Site Comparison *Note.* Purple points represent the same chimpanzees sampled from the first site (Figure 2), and orange points represent a second sample of chimpanzees.

To create a replication study, we repeated this process, taking another random sample of 10 chimpanzees from a different site. This sample is plotted in Figure 3 alongside our first sample, creating a within‐species (or experimental unit) replication, which could also be framed as a between site comparison, or a test of generalizability across sites.

The second sample of chimpanzees, in orange, had smaller neophobia and larger self-control scores than the first sample, in purple. Performing a two-sided Welch’s *t* test, both differences were statistically significant, *p*_neophobia_ < .001 and *p*_self-control_ = .04. This reflects the real variation between the sites, which were simulated at 28% for neophobia, and 14% for self-control. Our samples of just 10 animals captured this difference relatively accurately, estimating the group differences as 31% for neophobia and 14% for self-control. While our two samples provided good estimates of the true between-sample differences, our samples were poorly representative of the overall population of chimpanzees. Site 1 (purple), overestimated neophobia by 14% and self-control by 3%, whereas Site 2 underestimated neophobia by 17%, and self-control by 11%.

Having simulated a within-species replication, we proceeded to simulate a typical between-species comparison. To achieve this, we randomly sampled from the set of 100 animals at 100 sites, but this time of bonobos. All of the parameters determining bonobo behavior were kept the same as with the chimpanzees, except that we set the bonobo neophobia scores to be, on average, just under one standard deviation higher than the chimpanzee neophobia scores (specifically, this was set as the species difference being 1.5 times larger than the between-site standard deviation, such that:

$$\begin{array}{l} {\text{Neophobia}}_{\text{bonobo}}\\ \beta_{0}=950\\ L_{0l}∼N(0,100)\\ S_{0s}∼N(0,100)\\ e_{ls}∼N(0,50) \end{array}$$

**Figure 4:**
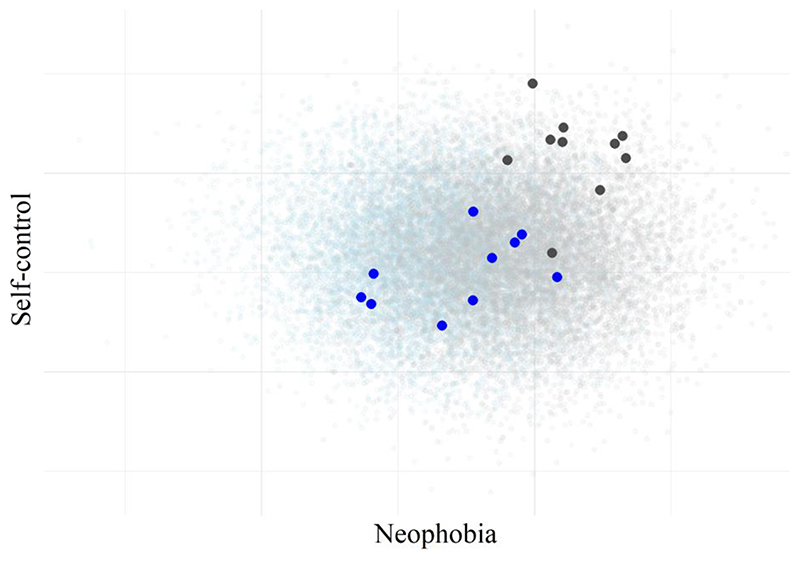
A Comparison Between Hypothetical Samples of Chimpanzees and Bonobos *Note.* Populations of 10,000 chimpanzees (light blue) and 10,000 bonobos (gray) sampled from 100 simulated sites. Samples of 10 chimpanzees and 10 bonobos from a single site are overlaid for chimpanzees (blue) and bonobos (dark grey).

The decision to make bonobos more neophobic than chimpanzees was arbitrary, and most empirical data supports the opposite conclusion (e.g., Forss et al., 2019). The average self-control scores were kept the same between species. Just as with the replication, we simulated all 10,000 chimpanzees and bonobos, and selected a site at random from which we sampled 10 chimpanzees, and a random site from which we sampled 10 bonobos. Figure 4 shows the results: the entire population of 10,000 chimpanzees in grey circles and 10,000 bonobos in blue circles, and our samples are highlighted.

Our samples in Figure 4 captured the direction of the population difference in neophobia scores, which were statistically significantly larger in the bonobo sample than the chimpanzees, *p*_neophobia_ < .001. However, the magnitude of this effect was overestimated by 41%. For self-control, where no population differences were simulated, our samples produced a statistically significant difference between chimpanzees and bonobos (*p*_self-control_ < .001), incorrectly estimating a species difference of over 40%. This highlights how even when a statistically significant difference is observed between species at different sites, it does not mean that the difference should be attributed to species identity alone. To explore this further, we investigated how often our comparison would return a statistically significant difference between the neophobia scores and self-control scores of our chimpanzee and bonobo samples. Because our simulation specified that there were no true differences between the species in self-control, this can provide our base-rate of false positive results, under the assumption that statistically significant results would be taken as evidence for a species difference. We simulated 100,000 comparisons between samples of 10 chimpanzees and 10 bonobos, each taken from a new site.

**Figure 5:**
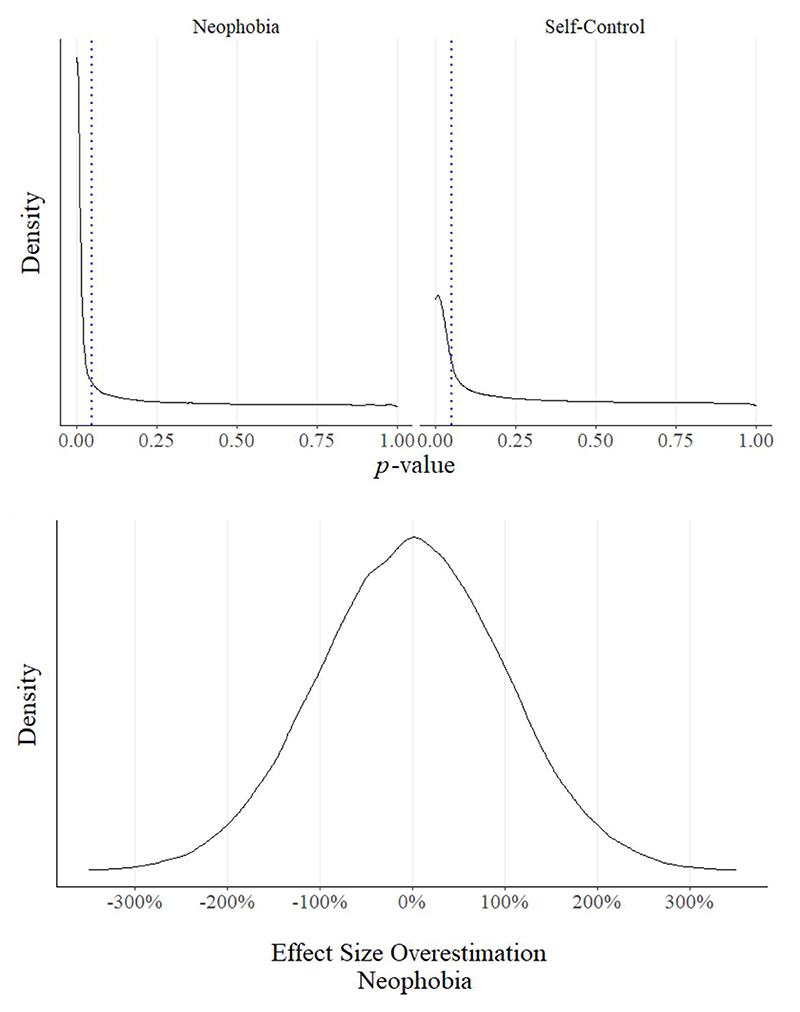
p-value Distributions and Effect Size Overestimation from Two Simulated Comparisons *Note.* Upper panel: *p*-value density distributions of two-sample *t*-tests from 100,000 comparisons between 10 hypothetical chimpanzees and 10 hypothetical bonobos, sampled at different sites. The simulation included between-site variation, and a species difference in neophobia, but not self-control. Lower panel: The density distribution of effect size overestimation for the 100,000 comparisons of neophobia behavior. No data are shown for self-control as the set difference was 0, therefore it was not possible to calculate the % overestimation for simulations with non-zero differences.

Across the 100,000 simulated comparisons, our small sample design detected a true difference between chimpanzees and bonobos in neophobia 66% of the time with alpha = .05, which looks quite promising. However, the 100,000 simulations also detected a difference between the chimpanzees and bonobos on the self-control measure 49% of the time, in which there were no species differences specified. Figure 5 (upper panel) plots the *p*-value distributions of the two comparisons, and the similarity between these distributions shows that observing a statistically significant difference between two samples, even if *p* < .05, is not necessarily strong evidence of an overall species difference. Figure 5 (lower panel) displays the degree of over- and under-estimation of the neophobia effect size across all samples. Strikingly, in 32% of comparisons, the effect size was overestimated or underestimated by over 100%.

### Strong and Weak Comparisons

Non-representative sampling leads to weak comparisons, and these comparisons are particularly troublesome when:

- There is a large ratio of within-species variation to between-species variation (MacLean et al., 2012), and absolute species differences are small. Such a scenario will mean the direction and magnitude of differences between samples will be volatile.
- Experimental units are not tested across samples of the same relative settings, measurements, and treatments, and because of this, measurement techniques systematically differ between research programs; For example, when a single population of experimental units is repeatedly sampled, or the same researchers and research groups perform most of the research, with the same experimental designs (Clark et al., 2019; Ioannidis, 2012a; Leavens et al., 2019). This could lead to highly replicable – within narrow boundary conditions - differences between samples being recorded, but these differences being a consequence of specific local features (often confounders) rather than general species differences.

In contrast, strong between-group comparisons should fulfill the following three criteria:

1) The results are consistent within experimental units across times, experimenters, treatments, and measurements within the claims’ boundary conditions.
2) The samples of experimental units being compared are tested from within the same relative populations of settings, treatments, and measurements relative to the claim.
3) The between-group differences can be replicated when resampling from the target populations of experimental units.

### Improving Sampling in Animal Research

There are several methods researchers can use to assess and model the effects of biased sampling on the reliability and generalizability of their research findings, which we have divided into experimental design and statistical methods.

#### Experimental Design

##### Increasing Heterogeneity

Increasing heterogeneity is a direct method of increasing the representativeness of a sample to a target population. By sampling more diversely from within the populations specified by a theory or claim, researchers can better estimate the population parameters of interest (Milcu et al., 2018; Voelkl et al., 2018, 2020; von Kortzfleisch et al., 2020). This could involve sampling from multiple sites, such as in large collaborative studies (Crabbe et al., 1999; Culina, Adriaensen et al., 2020; Many Primates, Altschul, Beran, Bohn, Call et al., 2019), but also by using multiple different experimenters and varying the conditions and treatments within sites (Baribault et al., 2018; Richter et al., 2010; Würbel, 2002). As an example, Rössler et al. (2020) compared the ability of a sample of wild-caught Goffin’s cockatoos and a sample of laboratory-housed Goffin’s cockatoos to physically manipulate an apparatus to access a reward. However, rather than presenting the cockatoos with a single apparatus, they were tested in an area with a total of 20 apparatuses. Because Rössler et al. sampled from a diverse range of treatments, we can be confident that - at least for these samples of cockatoos – the results are robust across variations in treatment. An ideal experiment might generate diverse samples across all feasible factors – sites, treatments, experiments, times of day, measurements etc., which will increase the replicability and generalizability of the results (Würbel, 2000); however, it is high-cost (Davies & Gray, 2015; Mook, 1983; Schank & Koehnle, 2009).

##### Increasing Homogeneity and Control

In contrast to increasing heterogeneity, a lower-cost approach is to increase standardization and control. For example, performing experiments with blinded experimenters only is more homogeneous than performing experiments with a mixture of blinded and non-blinded experimenters. From the re-sampling perspective, blinded and unblinded experimenters come from different populations, and most theories in comparative cognition make predictions that are independent of experimenter bias (i.e., do not predict that experimenter effects are essential for their predictions to be true). Similarly, homogeneity can be useful when a theory is most effectively tested within a subset of the populations that it might apply to. For example, animals are often trained before being tested when researchers attempt to isolate individual psychological mechanisms, such as learning. Such researchers are not usually interested in measuring variability due to neophobia or novel-object exploration, and so animals are familiarized with and trained on the task set-up before being tested to avoid including this “noise” in the dataset. The training pulls all individuals towards their theoretical maximum, increasing statistical power and the relevance of the collected data to the theory in question (Schank & Koehnle, 2009; Smith & Little, 2018), and this can increase the validity of between-group comparisons when the groups have markedly different learning histories (Leavens et al., 2019).

##### Statistical Approaches: Multilevel Models, Phylogenetic Models, and Being Cautious

The variation in experimental units, settings and measurements can be modeled statistically, using multilevel models (e.g., DeBruine & Barr, 2019; McElreath, 2016), and these should include phylogenetic information for multi-species datasets (Cinar et al., 2020; Davies et al., 2020; Freckleton, 2009; MacLean et al., 2012; Stone et al., 2011). Perhaps most useful are these models’ ability to pool information across species and shrink extreme species estimates towards the mean response for a given clade, but it also has the benefit of more closely aligning research fields with evolutionary theory (MacLean et al., 2012; Vonk & Shackelford, 2012). However, generating appropriate multilevel or evolution-informed models of animal behavior is a complex task, which will require a decent amount of data and knowledge about how traits may have been selected. Often, these data and this knowledge will not be available.

When researchers cannot introduce or model variation in their designs, they are faced with a dilemma. Uncertainty intervals will be too narrow with respect to the researcher’s populations of interest, but the researcher has no direct means of estimating by how much. One solution is for researchers to artificially increase the uncertainty in their statistical estimates (Kafkafi et al., 2017; Yarkoni, 2019), and this could be informed by data on the ratio of between-site to between-species variance from similar multi-site studies; however, this introduces a trade-off between statistical power and false positive discovery rates. In general, researchers should be cautious when interpreting extreme results observed from single samples, such as the 80% great tit performance on the cylinder task we saw earlier, which regressed to around 40% upon resampling.

##### Barriers

Concerns about replicability and representativeness have surfaced often in animal behavior and cognition research, at a variety of levels (Beach, 1950; Beran, 2012; Bitterman, 1960; Boesch, 2012, 2020; Brosnan et al., 2013; Clark et al., 2019; Dacey, 2020; Eaton et al., 2018; Farrar et al., 2020; Janmaat, 2019; Leavens et al., 2019; Schubiger et al., 2019; Stevens, 2017; Szabó et al., 2017; van Wilgenburg & Elgar, 2013; Vonk, 2019). However, it is unclear whether any real progress has been made towards understanding the prevalence and consequences of low representativeness in these fields, and we suggest that there are four main reasons why, which are theoretical, practical, motivational, and educational (see also Farrar & Ostojić, 2020).

First, *theoretically*, researchers may believe that their samples are representative of their target populations, or that if they are not, that this does not heavily impact the validity of their results. Such a position may be justifiable, for example when, i) relatively independent animals can be sampled by the same team (e.g., with dog research, or serially captured and released samples), ii) animals are highly trained (Leavens et al., 2019; Skinner, 1956; Smith & Little, 2018), iii) unique case studies, and iv) when heterogenization is used. However, if researchers do justify the generalizability of their findings theoretically, then these arguments should be made explicitly within papers (Simons et al., 2017), be solicited by editors and reviewers (Webster & Rutz, 2020), or provided as commentaries on entire research programmes. These justifications will be strongest when they employ a sampling approach to experimental design, and do not excessively focus on the experimental unit over other levels of sampling variance (Farrar & Ostojić, 2020).

Second, researchers may not *practically* have access to the resources needed to test the representativeness of their samples. They may only have one sample, and other laboratories with access to the same species might not exist. This is a problem – it may not be possible to study hard-to-reach samples in a reliable or replicable manner (Lange, 2019; Leonelli, 2018). However, researchers with such samples can take steps to ensure that their results are as robust as possible, and that an appropriate amount of uncertainty is disclosed, through the experimental and statistical techniques we have mentioned in this article, and so practical constraints do not inherently bar researchers from addressing issues of representativeness.

Third, researchers may lack the *motivation or incentives* to test the representativeness of their samples, and the stability of their results across experimental units, settings, treatments, and measurements. If the scientific incentive and funding structure selects for compelling narratives, oversold findings, and ground-breaking results (Higginson & Munafò, 2016; Ioannidis, 2012b; Smaldino & McElreath, 2016) over rigor, self-correction and understanding, the comparative researcher who attempts to replicate their findings across experimental units and settings may be disadvantaged in terms of common scientific metrics (citations and publications). Addressing these incentive problems is a large task which requires action at the level of the individual (Yarkoni, 2018), organization (Nosek et al., 2012) and society (Amann, 2003; Lazebnik, 2018). Encouragingly, there appears to be a desire to perform more replication studies, in some fields. Fraser et al. (2020), for example, surveyed 439 ecologists, and found that researchers thought replications are very important, reflect a “crucial” use of resources, and should be published by all journals.

Fourth, researchers may be unaware or have not accessed the *education* needed to effectively consider and model sampling variability in their studies. Statistical misconceptions (Goodman, 2008; Hoekstra et al., 2014) and mis-practice (Hoekstra et al., 2012; Nieuwenhuis et al., 2011) are prevalent, present in textbooks (Price et al., 2020), and are perhaps only more likely with the increasing complexity of statistical procedures and software that are available (Forstmeier & Schielzeth, 2011; Schielzeth & Forstmeier, 2009; Silk et al., 2020). At the same time, many university programmes may lack teaching on replication related topics (TARG Meta-Research Group, 2020), and there are no requirements to continue education for researchers following formal qualifications, i.e., post PhD, and neither has considering the replicability or generalizability of findings been well integrated with much of the publishing system (Neuliep & Crandall, 1990, 1993; Webster & Rutz, 2020).

These four barriers will be effectively combatted by top-down measures, such as funding bodies and institutions signing initiatives like the San Francisco Declaration on Research Assessment (DORA), and providing contracts and the job-security needed to promote researchers’ scientific development over output metrics. However, bottom-up initiatives from within animal behavior research could be effective and are at least under researchers’ direct control (Yarkoni, 2018), and individuals can address each of the four barriers above by, and helping others in, i) discussing how their sampling plans relate to their research aims, and describing what these research aims are, ii) discussing the ethical and practical constraints on diversifying their sampling plans if this is desirable, and considering changes to research designs and generating collaborations if the benefits could outweigh the costs, iii) examining their own motivations when performing science and publishing research findings and, iv) actively pursuing further education in research design and statistical analyses.

## Conclusions

In this article, we applied a resampling definition of replication to animal cognition, and we explored the consequences of small and non-independent (poorly representative) samples in animal behavior and cognition research. Limited sampling is likely a large constraint on the replicability and generalizability of research findings, and it has particularly concerning implications for the accuracy of between group or between species inferences. Comparative researchers should be especially concerned about a lack of representativeness of their samples when there is a large ratio of within-species variation to between‐species variation, and when the same researchers, animals, and research methods are used repeatedly. Finally, we discussed how researchers can use techniques such as heterogenization, homogenization, and statistical modeling to improve the replicability and representativeness of their results, and considered the practical, theoretical, and motivational factors that might prevent a full assessment of reliability and representativeness in the field.

## Figures and Tables

**Table 1:** A Resampling Account of Replication (Adapted from Machery, 2020)

An experiment samples from:	A replication *resamples* from:
A population of experimental units, e.g., a population of a species in captivity	The same population of experimental units
A population of treatments, e.g., experimental conditions	The same population of treatments
A population of measurements, e.g., definitions of success on a trial	The same population of measurements
A population of settings, e.g., sites and times	The same population of settings

**Table 2:** Results and Claims from Four Species Tested on the Cylinder Task

Study	Group	Score	Claim
Ferreira et al., 2020	High ranging chickens	24%	“High rangers had the worst performance of all species tested thus far” (p. 3)
	Low ranging chickens	40%	
Isaksson et al., 2018	Great tits	80%	“The average performance of our great tits was 80%, higher than most animals that have been tested and almost in level with the performance in corvids and apes.” (p. 1, abstract)
Langbein, 2018	Goats	63%	“The results indicated that goats showed motor self-regulation at a level comparable to or better than that of many of the bird and mammal species tested to date.” (p. 1, abstract)
Lucon-Xiccato et al., 2017	Guppies	58%	“A performance fully comparable to that observed in most birds and mammals” (p. 1, abstract)
